# A Systematic Review on the Optimal Dose and Duration of Ready-to-Use Therapeutic Food (RUTF) for 6–59-Month-Old Children with Severe Wasting or Oedema

**DOI:** 10.3390/nu15071750

**Published:** 2023-04-03

**Authors:** Blessings H. Likoswe, Bernadette Chimera-Khombe, Noel Patson, Apatsa Selemani, Isabel Potani, John Phuka, Kenneth Maleta

**Affiliations:** 1 Department of Nutrition and Dietetics, School of Global and Public Health, Kamuzu University of Health Sciences, Private Bag 360, Chichiri, Blantyre 312225, Malawi; blikoswe@medcol.mw (B.H.L.); chimerab@iarc.who.int (B.C.-K.); npatson@medcol.mw (N.P.); aselemani@medcol.mw (A.S.); isabel.potani@sickkids.ca (I.P.); jphuka@kuhes.ac.mw (J.P.); 2 Translational Medicine Program, Hospital for Sick Children, Toronto, ON M5G 1X8, Canada; 3 Department of Nutritional Sciences, Faculty of Medicine, University of Toronto, Toronto, ON M5S 1A1, Canada

**Keywords:** severe acute malnutrition, ready-to-use therapeutic food, systematic review

## Abstract

The World Health Organisation (WHO) recommends that severe wasting and/or oedema should be treated with ready-to-use therapeutic food (RUTF) at a dose of 150–220 kcal/kg/day for 6–8 weeks. Emerging evidence suggests that variations of RUTF dosing regimens from the WHO recommendation are not inferior. We aimed to assess the comparative efficacy and effectiveness of different RUTF doses and durations in comparison with the current WHO RUTF dose recommendation for treating severe wasting and/or oedema among 6–59-month-old children. A systematic literature search identified three studies for inclusion, and the outcomes of interest included anthropometric recovery, anthropometric measures and indices, non-response, time to recovery, readmission, sustained recovery, and mortality. The study was registered with PROSPERO, CRD 42021276757. Only three studies were eligible for analysis. There was an overall high risk of bias for two of the studies and some concerns for the third study. Overall, there were no differences between the reduced and standard RUTF dose groups in all outcomes of interest. Despite the finding of no differences between reduced and standard-dose RUTF, the studies are too few to conclusively declare that reduced RUTF dose was more efficacious than standard RUTF.

## 1. Introduction

Severe Acute Malnutrition (SAM), diagnosed by weight-for-height z-scores (WHZ) below −3 SD and/or mid-upper arm circumference (MUAC) below 11.5 cm and/or nutritional oedema, globally affects 17 million children under-five and increases their risk of morbidity, mortality and developmental delay [1,2]. SAM is managed in two phases, namely stabilisation (for those with medical complications) and rehabilitation. During stabilisation in inpatient facilities, SAM cases are treated for their clinical complications, including severe oedema, infections, and loss of appetite, and feeding is re-established using therapeutic milk, F75 and F100 [3]. Rehabilitation is then achieved using ready-to-use therapeutic food (RUTF) to achieve catch-up growth, and this is usually outpatient-based.

The World Health Organisation (WHO) recommends an RUTF intake of 150–220 kcal/kg/day for 6–8 weeks with the aim of achieving a weight gain velocity of 5–10 g/kg/day [4]. This is extrapolated from inpatient settings where the provision of 165 kcal/kg/day, provided through structured supervised feeds, given several times per day, and ad libitum provision of family foods has achieved rapid weight gains, up to 15.6 g/kg/day [5]. However, in most treatment programs, the average outpatient rates of weight gain are usually lower, below 6 g/kg/day, resulting in treatment durations that range from 5 to 16 weeks to attain recovery and program treatment failure and relapse rates of 30% and 37%, respectively [6,7,8]. Besides the dose of RUTF, the adequacy of therapeutic feeding protocols may also be affected by the background prevalence of infection/inflammation and dietary intakes, which may render current quantities inadequate or excessive at some stages of treatment depending on context.

The quantity and duration of treatment of RUTF are important elements for the improved treatment outcomes of SAM in children. These considerations have recently spurred research and program modifications that have resulted in dose reduction, optimisation or increases to improve outcomes and reduce the duration of treatment while keeping costs low. This systematic review thus aimed to assess the comparative efficacy and effectiveness of different RUTF doses and durations in comparison with the current WHO RUTF dose recommendation in treating severe wasting and/or oedema among 6–59-month-old children.

## 2. Materials and Methods

We conducted a systematic review and meta-analysis in accordance with the Preferred Reporting Items for Systematic Reviews and Meta-analyses (PRISMA) guidelines [9]. A protocol for this systematic review was registered in the International Prospective Register of Systematic Reviews (PROSPERO) [CRD4202127675] and can be accessed https://www.crd.york.ac.uk/prospero/display_record.php?RecordID=276757.

A systematic electronic search was conducted by a research librarian (AS) on 9 September 2021 and 28 September 2021 to identify published relevant articles in major international databases from 1946 to the present. Medical subject headings and free text words covering the following concepts were used: ready-to-use therapeutic food, RUTF, plumpy nut or yum plum, and infant or preschool child, as comprehensively shown in Supplementary Materials Table S1. The databases searched included: MEDLINE (Ovid), Embase (Ovid), CINAHL (EBSCOhost), Web of Science (Clarivate Analytics), and Cochrane Library. Other databases searched were, Global index Medicus (LILACS, African Index Medicus, IMEMRx, IMSEAR, WPRO) to specifically cater to low and middle-income countries (LMICs) and evidence that may have been missed in the other databases. Furthermore, a search for both completed and ongoing trials was conducted in the following databases: WHO International Clinical Trials Registry Platform (ICTRP) (http://www.who.int/ictrp/en/, accessed on 28 September 2021), ClinicalTrials.gov, and Epistemonikos (https://www.epistemonikos.org, accessed on 28 September 2021), Conference Proceeding Citation Index (CPCI) and Cochrane Central Register of Controlled Trials (CENTRAL. We also searched for grey literature and more information from the Emergency Nutrition Network’s website (https://www.ennonline.net/, accessed on 28 September 2021) and the state of acute malnutrition website (https://www.acutemalnutrition.org/, accessed on 28 September 2021). Citation tracking was also employed by going through references of identified studies and Google Scholar and other similar databases. We did not restrict our searches by language or publication status. Covidence software was used for screening the studies and removal of duplicates [10].

Inclusion criteria were individually or cluster-randomised controlled trials as well as non-randomized controlled studies that were controlled before-after (CBA), interrupted time series (ITS) and repeated measures. The age group of interest was children aged 6–59 months with severe wasting and/or oedema, which was defined as [3] weight-for-height/length z-scores (WHZ/WLZ) below −3 standard deviations and/or mid-upper-arm-circumference (MUAC) of <11.5 cm and/or bilateral pitting oedema +/++/+++. The intervention of interest was described as varying quantity or duration of RUTF, in comparison to what is currently recommended, which could include a lower or higher quantity of RUTF, defined as RUTF below 150 kcal/kg/day or above 220 kcal/kg/day, and/or RUTF provided for a shorter or longer duration defined as RUTF treatment for equal to or less than 6 weeks or over 8 weeks. The comparator of interest was described as a RUTF dose between 150–220 kcal/kg/day provided until recovery is achieved, referred to in this report as ‘standard dose’. Six subgroup analyses were also planned as follows.

1. Age groups 6–24 months and >24–59 months.
2. A comparison of treatment settings, i.e., RUTF initiated in outpatient/community care versus RUTF initiated during inpatient care.
3. Comorbidity status, i.e., with or without comorbidities.
4. HIV status, i.e., HIV positive on antiretroviral treatment versus HIV positive not on antiretroviral treatment versus HIV negative.
5. WLZ or WHZ below −3 standard deviations and/or MUAC of <11.5 cm and/or bilateral pitting oedema +/++/+++
6. Region (Africa, South Asia, South-eastern Asia and Oceania)

There were seven outcomes of interest for this, namely, anthropometric recovery, anthropometric outcomes, sustained recovery, time to recovery, non-response, mortality, and readmission, which are described in Supplementary Materials Table S2. All non-intervention studies, non-human studies and studies examining only infants and children with moderate wasting were excluded. Furthermore, studies that did not report at least one of the outcomes of interest were not included.

Two reviewers (BCK and BHL) independently screened the studies to identify those which met the inclusion and exclusion criteria and any contradictions, which occurred during the screening process, were resolved by a third reviewer (IP or KM). Summary estimates from the studies were extracted independently and in duplicate by the three reviewers (BCK, BHL, IP) in a Research Electronic Data Capture (REDCap) database. For outcomes of interest, which were not reported in the studies (but were assumed to be available), inquiries were made directly to the authors for their provision.

A quantitative synthesis of the data using meta-analysis based on the seven outcomes of interest was undertaken in Stata 16.1 (StataCorp, College Station, TX, USA). Pairwise and random-effect meta-analyses were performed to compare the effect of varying RUTF doses and dose durations on the respective outcomes of the currently recommended RUTF dose (i.e., 150–220 Kcal/kg/day). The effect size heterogeneity between the studies was assessed using *Q*^2^, *T*^2^ and *I*^2^. To accommodate effect size variation from study to study, the meta-analysis was performed using a random-effects model. We used the random effects model because we assumed that the observed estimates of treatment effect could vary across studies because of real differences in the treatment effect in each study as well as sampling variability (chance). Forest plots were also used to illustrate the effect estimates for the meta-analyses. Intention-to-treat (ITT) results were used in all the analyses. Effective sample sizes were computed for cluster-randomized controlled data based on the number of clusters, mean number of participants per cluster, and the intra-cluster correlation coefficient of 0.015 from a similar study in children with severe wasting in an outpatient setting [11].

## 3. Results

**Figure 1:**
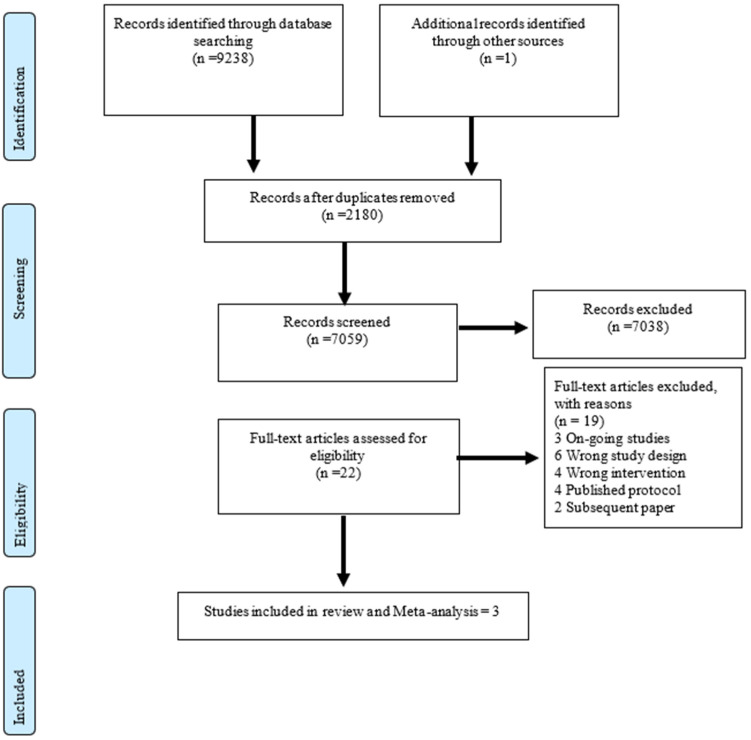
PRISMA flow chart indicating included and excluded studies and the overall reasons for exclusion in a systematic review of varying quantities and duration of RUTF for the treatment of severe wasting and/or oedema in children. Note: Reasons for the exclusion of studies are presented comprehensively in Supplementary Materials Table S4.

A total of 9238 studies were imported into Covidence for screening, 2180 duplicates were removed, and 7058 studies remained (Figure 1). After title and abstract screening, 7038 studies were deemed irrelevant, leaving 21 studies that were screened in full. After the full-text screening, three studies were deemed eligible for data extraction and analysis. All three studies were included in at least one meta-analysis of the outcomes of interest. The search and screening identified three ongoing studies (Supplementary Materials Table S3). The list of excluded studies and main reasons are presented in detail in Supplementary Materials Table S4.

Three studies were included, and their detailed characteristics are reported in Table 1. Outcomes such as a weekly change in MUAC that was included in all three papers were assessed in a total of 2119 participants. Although other outcomes, such as mean MUAC and mean weight at discharge, were only included in two papers and thus had smaller sample sizes. In brief, The first included study was a randomised control trial in Burkina Faso [12]. The study aimed at assessing the non-inferiority of weight gain velocity of children with severe wasting receiving a standard RUTF dose for two weeks, followed by a reduced dose thereafter (reduced), compared with a standard RUTF dose throughout the treatment. The study had a non-inferiority margin fixed at −0.5 g/kg/day for their primary outcome, weight gain velocity.

The second included study was an open-label, randomised non-inferiority trial that was conducted in the Democratic Republic of Congo (DRC) [13]. The study aimed to assess whether an integrated acute malnutrition strategy using gradually decreasing doses of RUTF while weight and MUAC increased was non-inferior to the current standard of care. The primary endpoint was the proportion of recovery among children with severe acute malnutrition measured throughout the 6-month follow-up period of the trial. The non-inferiority margin was fixed at 10% and was planned to compare the reduced dose and standard groups in terms of recovery proportion over the 6-month follow-up trial period, using both the intent-to-treat (ITT) and per-protocol (PP) approaches.

**Table 1:** Characteristics of studies included in a systematic review of varying doses and dose duration of RUTF for the treatment of severe wasting and/or oedema in children aged 6–59 months.

Study	Setting	Study Design	Baseline Sample Size	Study Population	Dose Description	Outcomes
Kangas et al. [12]	Burkina Faso (low-income)	Randomised non-inferiority trial. Block randomisation (block sizes from 2 to 8) was stratified at the health centre level, and a total of 10 health centres were included as study sites between both arms.	Intervention: 402 Comparison: 399	Age6 –59 months Phenotype Severe wasting defined as WHZ < −3 and/or MUAC < 11.5 cm and no oedema	Reduced dose RUTF group Week 1–2 Standard RUTF dose based on the categorised weight of the child (144–214 kcal/kg/day) Week 3–16 reduced RUTF dose based on the categorised weight of the child (67–167 kcal/kg/day) Standard-dose RUTF group Week 1–16 Standard RUTF dose based on the categorised weight of the child (144–214 kcal/kg/day)	Anthropometric recovery ^1^ Anthropometric measures and indices (weight, weight gain velocity ^2^, MUAC, MUAC gain velocity, HAZ, WHZ, WAZ) Sustained recovery Time to recovery ^3^ Length of stay ^4^ Non-response ^5^ Mortality Relapse ^6^ False discharge ^7^
Cazes et al., [14]	The Democratic Republic of Congo (low-income)	Non-inferiority (10% margin), individually randomised controlled trial. Block randomisation was used, and children were assigned 1:1 to a study arm by the randomisation software. Four health centres were included.	Intervention: n = 242 Comparison: n = 240	Age 6–59 months Phenotype severe acute malnutrition children with MUAC <11.5 cm or a WHZ <−3 or bipedal oedema.	Intervention group RUTF dose was relative to body weight and MUAC gradually decreased as the child’s weight, and MUAC increased. MUAC < 11.5 cm: RUTF 170–200 kcal/kg/day MUAC ≥ 11.5 cm and oedema: RUTF 170–200 kcal/kg/day (until oedema was resolved) MUAC between 11.5 cm and 11.9 cm: RUTF ≥ 125–190 kcal/kg/day MUAC ≥12.0 cm: RUTF 50–166 kcal/kg/day Standard dose group RUTF dose is based on the current WHO recommendations. RUTF 150–200 kcal/kg/day according to weight category Additional treatments for both groups Amoxicillin 50–100 mg/kg/day for 7 days Vitamin A and an anthelmintic (deworming treatment) A rapid malaria test was carried out at inclusion and artemisinin-based combination therapy was prescribed for positive cases.	Anthropometric recovery ^8^ Anthropometric parameters (Weight, Weight gain, Daily weight gain, Height, Height gain, MUAC, MUAC gain, Weekly MUAC gain, WHZ, WAZ, HAZ) Sustained recovery ^9^ 2. Time to recovery ^10^ 3. Non-response rate ^11^ 4. Mortality 5. Relapse ^12^ 6. Default rate
Maust et al. [14]	Sierra Leone	Cluster-randomised unblinded controlled clinical trial The unit of randomisation was the site of treatment, and subjects were not randomly assigned.	Intervention: 1100, SAM (n = 326) Comparison: 857, SAM (n = 537)	Age 6–59 months The phenotype in the intervention group SAM-MUAC < 11.5 cm or the presence of bipedal oedema (data for this group were obtained directly from the authors) MAM-MUAC > 11.4 and <12.5 cm The phenotype in the standard group SAM and MAM–Based on the prescribed malnutrition treatment protocol of the government of Sierra Leone.	Integrated management group SAM children received a ration of RUTF at 175 kcal/kg/d for 12 weeks. When children progressed from SAM to MAM, they received RUTF at 75 kcal/kg/d. Additionally, all children also received oral rehydration solution, malaria prophylaxis, and a program of immunisations that included the entire complement recommended by WHO (at discharge). The caregivers were referred to a peer-counselling care group that focused on child nutrition and health issues. Children who recovered were given 500 g of a lipid nutrient supplement that provided 100% of the RDA for all micronutrients and 200 kcal/d when taken as 40 g/d. Standard management group SAM children received RUTF at 200 kcal/kg/day, high-dose vitamin A, folic acid, amoxicillin, a dose of antimalarial drug, albendazole and measles vaccination until they fully recovered. Children who were recruited as MAM received Super Cereal Plus, a fortified blended flour containing some oil and milk powder, given at a ration of 1250 kcal/d, vitamin A, albendazole, iron and measles vaccination.	Anthropometric recovery ^13^ Anthropometric parameters (daily MUAC gain, daily weight gain, WHZ, Height, Height gain, HAZ, WAZ) Non-response ^14^ Died or referred to inpatient ^15^

The table includes all outcomes examined for each study. KANGAS: ^1^ Anthropometric recovery is defined as reaching a WHZ of −2 for those admitted with a WHZ < −3 only, or MUAC of 125 mm for those admitted with a MUAC < 115 mm only, or both WHZ −2 and MUAC of 125 mm for those admitted with both WHZ < −3 and MUAC < 115 mm upon two consecutive visits and absence of any illness. ^2^ Weight gain velocity-calculated from admission to discharge (primary outcome) and weight gain velocity calculated from admission to after two weeks (secondary outcome). ^3^ Time to recovery- the number of days spent from admission to recovery. ^4^ Length of stay -calculated as the number of days spent from admission to either recovery, referral, nonresponse, false discharge, or last visit before defaulting, lost to follow-up, or death. ^5^ Non-response-included children not reaching anthropometric discharge criteria by 16 weeks of treatment who were referred to inpatient care for further examinations. ^6^ Relapse- recorded over 12 weeks following recovery and were defined as presenting a WHZ < −3 and/or a MUAC < 115 mm, or any grade of bilateral oedema. ^7^ False discharges- included children who were erroneously discharged as recovered or referred, but upon analysis did not meet the criteria. CAZES: ^8^ Anthropometric recovery- MUAC ≥ 125 mm or WHZ ≥ −1.5. ^9^ Sustained recovery—Children alive without acute malnutrition no relapse at 6 months. ^10^ Time to the recovery-total length of time from admission to recovery. ^11^ Non-response—Acute malnutrition from inclusion to 6 months. ^12^ Relapse—defined as a MUAC < 115 mm or a WHZ < −3 or the presence of bilateral nutritional oedema after the child was free of acute malnutrition at a previous visit or after the child relapsed into moderate acute malnutrition and afterwards met the criteria for severe acute malnutrition. MAUST: ^13^ Anthropometric recovery—In the intervention arm, it was determined by MUAC > 12.4 cm and in the standard arm it was determined by WHZ of −2 or greater. ^14^ Non-response—Children who remained in SAM or MAM. ^15^ Died or referred to inpatient—After children were referred to inpatient, they were not followed up and it was assumed that most of them died in the hospital.

The third included study was a cluster-randomized controlled trial in Sierra Leone conducted in 10 centres, treating general acute malnutrition (GAM) in children aged 6–59 months old [13]. The study aimed to determine the recovery and coverage rates of GAM of an integrated protocol with a single food product, RUTF, compared with standard management. Every child was assigned to 1 of 4 mutually exclusive categorical outcomes at their final visit for acute care: recovered, remained malnourished, died or lost to follow-up. The primary outcomes were coverage and recovery rate, which were analysed on an intention-to-treat basis.

All studies reported intention-to-treat (ITT) and per-protocol (PP) results, but in this review, only ITT results are reported.

For Kangas et al. [12], the study duration was 16 weeks, and all outcomes were assessed at 16 weeks. The rate of weight gain and the rate of MUAC gain were also measured at 2 weeks. Post-discharge outcomes were based on a 12-week follow-up period post-recovery for all children that recovered. The study duration for Cazes et al. [14] was 6 months (24 weeks). Recovery rates were assessed at 12 weeks and 16 weeks, whereas all the other outcomes (including post-discharge) were assessed at 24 weeks from baseline. The study duration for Maust et al. [13] was 12 weeks. Children in the intervention group were followed up with every 2 weeks, whereas children in the standard arm group were followed up weekly. All endpoint outcomes were assessed at 12 weeks, and post-discharge outcomes in children who recovered were assessed 6 months after recovery. Only ITT results are used for the analyses in this review. Sub-group analyses could not be conducted because of limited studies.

**Figure 2:**
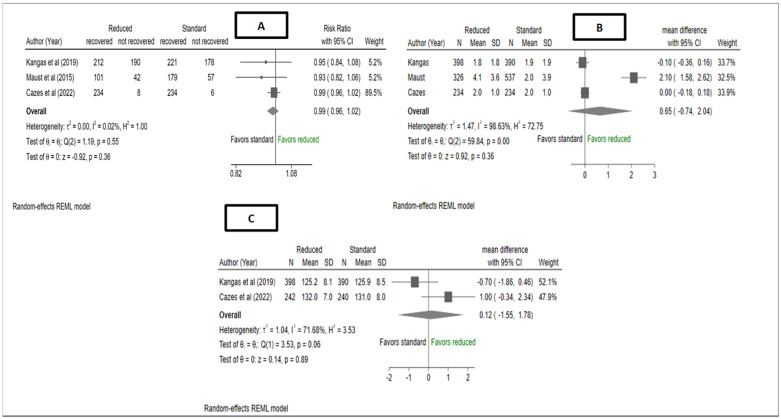
Forest plots of (**A**) anthropometric recovery rates, (**B**) weekly change in MUAC (from admission to discharge (Kangas et al., Maust et al. [12,13]) and at 6 months (Cazes et al. [14]) and (**C**) mean MUAC at discharge (Kangas et al. [12]) and 6 months (months (Cazes et al. [14]) from a meta-analysis of studies comparing reduced RUTF dose with standard RUTF dose for the treatment of severe wasting and/oedema. Effect estimates for Cazes et al. and Maust et al. (means and standard deviations) were obtained directly from the authors. Anthropometric recovery was assessed at 16 weeks for Kangas et al. and Cazes et al., whereas, for Maust et al., it was assessed at 12 weeks. Kangas et al. [12], defined anthropometric recovery as attaining either a WHZ of ≥−2 or a MUAC of ≥125 mm or both on two consecutive visits and the absence of any illness. Cazes et al. [14] defined anthropometric recovery as MUAC of ≥ 125 mm in the reduced RUTF dose group and MUAC of ≥125 mm or WHZ score of ≥−1.5 in the standard RUTF dose group. Maust et al. [13] defined anthropometric recovery as MUAC > 124 mm (intervention groups) and WHZ ≥ −2 (standard group). Recovery rates were assessed from admission to discharge in all studies. For Cazes et al., recovery is based on the standard definition applied to both study arms, which is a MUAC ≥ 12.5 cm or a WHZ ≥ 0.5. Kangas et al. [12] assessed the rate of MUAC gain at discharge, and Cazes et al. [14] assessed the rate of MUAC at six months. Data for Maust et al. [13] were reported as mm/day but were converted to mm/week. Mean MUAC in Cazes was assessed at six months, whereas for Kangas, it was assessed at discharge. Only ITT results were meta-analysed for both studies.

As shown in Figure 2, the pooled estimate of anthropometric recovery rate at 16 weeks showed no difference between reduced RUTF dose and standard RUTF dose, risk ratio (RR):0.99, 95% CI (0.96, 1.02), I^2^ = 0.02%, *p* = 0.36. The study by Cazes et al. [14] also reported children that reached anthropometric recovery (based on the standard definition of recovery in both study arms) by 12 weeks between the reduced RUTF dose arm 215/240 (89%) and standard RUTF arm 216/240 (90%) (*p* = 0.85).

The meta-analysis showed that there was no difference between the reduced RUTF dose group and the standard RUTF dose group in the rate of MUAC gain (mm/week). The mean difference across the studies was 0.62, 95% CI (−0.74, 1.98), I^2^ = 98.35%, *p* = 0.37. Another pooled finding from the meta-analysis did not find any difference in mean MUAC (cm) at recovery between the reduced RUTF dose and the standard RUTF dose arms (Figure 2) MD: 0.12, 95% CI (−1.55, 1.78), I^2^ = 71.68%, *p* = 0.89. However, Cazes et al. [14] further reported differences in the proportion of children who had MUAC < 12.5 (wasting) at 6-months-follow-up, which were 0/242 (0%) and 71/240 (30%) in the reduced RUTF dose group and standard RUTF dose groups respectively (*p* < 0.0001). Additionally, this study also reported no differences in the proportions of children with MUAC < 11.5 (severe wasting) between the reduced RUTF dose groups 5/154 (3%) and standard RUTF dose groups 4/158 (3%), *p* = 0.75 at 6-month follow-up. Kangas et al. [12] also reported a mean rate of weekly MUAC gain (mm/week) that was assessed from after week 2 of treatment to discharge, and there were no differences between the reduced and standard-dose RUTF groups at 1.1 ± 1.7 and 1.4 ± 1.9, respectively, with an unadjusted MD: −0.2; 95% CI (−0.5, −0.00), *p* = 0.05.

**Figure 3:**
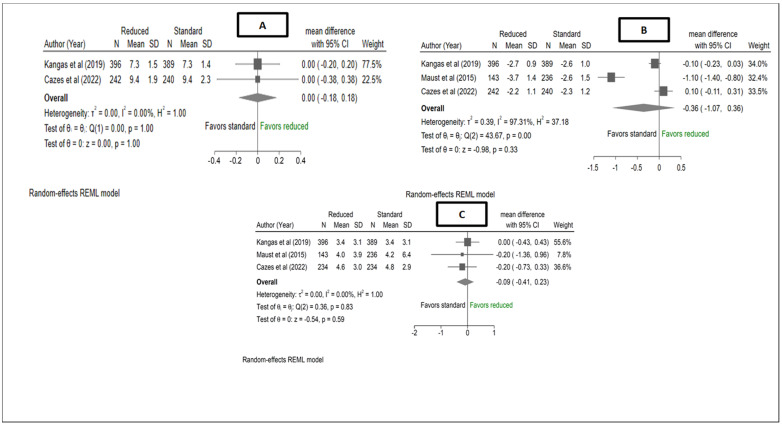
Forest plots of (**A**) mean weight and (**B**) rate of weight gain from admission to discharge (Kangas et al. Maust et al. [12,13]) and at 6 months (Cazes et al. [14]), and (**C**) mean change in WAZ at recovery from a meta-analysis of studies comparing reduced RUTF dose with standard RUTF dose for the treatment of severe wasting and/oedema. Effect estimates for Cazes et al. (means and standard deviations) were obtained directly from the authors. Mean weight in Cazes was assessed at six months, whereas for Kangas, it was assessed at discharge. Mean WAZ in Kangas et al. [12] was assessed at recovery whereas, for Cazes et al. [14] it was assessed at 6 months from baseline. Only ITT results were meta-analysed, for both studies.

There were no differences in mean weight (kg) at discharge between the reduced RUTF dose and standard RUTF dose (Figure 3), MD: 0.00, 95% CI: (−0.18, 0.18), I^2^ = 0%, *p* = 1.00). The pooled estimate also showed no difference in the mean rate of daily weight gain (g/kg/day) between reduced RUTF dose and standard RUTF dose in treating (from admission to discharge) severe wasting and/or oedema, with an MD of −0.09, 95% CI (−0.41, 0.23), I^2^ = 0%, *p* = 0.59. Additionally, Kangas et al. [12] reported a difference in the mean rate of daily weight gain of −0.4 g/kg/day, 95% CI (−0.8, −0.02), *p* = 0.04 between the two study arms, after the first two weeks of treatment. The reduced arm had a mean (mean ± SD) rate of daily weight gain of 2.3 ± 2.6, whereas the standard RUTF dose arm had a mean weekly weight gain rate of 2.7 ± 2.9. A pooled estimate showed no difference between the reduced RUTF dose and the standard RUTF dose in mean change in WAZ at recovery, MD −0.36, 95% CI (−1.07, 0.36), I^2^ = 97.31%, *p* = 0.33 (Figure 3).

**Figure 4:**
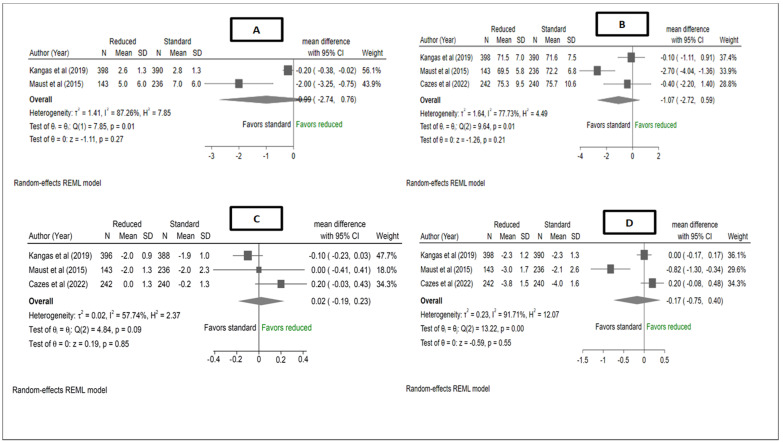
Forest plot of (**A**) mean height, (**B**) rate of height gain, (**C**) mean change in HAZ, and (**D**) WHZ at recovery (Kangas et al. and Maust et al. [12,13]) and 6 months (Cazes et al. [14]) from a meta-analysis of studies comparing reduced RUTF dose with standard RUTF dose for the treatment of severe wasting and/oedema. Effect estimates for Cazes et al. and Maust et al. (means and standard deviations) were obtained directly from the authors. Mean height in Kangas et al., and Maust et al. [12,13]. were assessed at recovery, whereas, for Cazes et al. [14], it was assessed at 6 months. Mean HAZ in Kangas et al. [12] was assessed at recovery whereas, for Cazes et al. [14], it was assessed at 6 months from baseline. Mean WHZ in Kangas et al., and Maust et al. [12,13] was assessed at recovery, whereas, for Cazes et al. [14], it was assessed at 6 months from baseline. Only ITT results were meta-analysed, for all studies.

The pooled estimate for absolute height at recovery showed no difference between reduced RUTF dose and standard RUTF dose in mean height (cm), MD −1.07, 95% CI (−2.72, 0.59), I^2^ = 77.73%, *p* = 0.21 (Figure 4). A pooled estimate of the two studies which assessed the rate of height gain showed no difference between reduced RUTF and standard RUTF in the rate of height gain, MD: −0.99, 95% CI (−2.74, 0.76), I^2^ = 87.26%, *p* = 0.27. There was no difference between reduced RUTF dose and standard RUTF dose in mean change in HAZ at recovery, MD −0.17, 95% CI (−0.75, 0.40), I^2^ = 91.71%, *p* = 0.55. The pooled estimate of WHZ at recovery showed no difference between reduced RUTF dose and standard RUTF dose, MD 0.02, 95% CI (−0.19, 0.23), I^2^ = 57.74%, *p* = 0.85.

**Figure 5:**
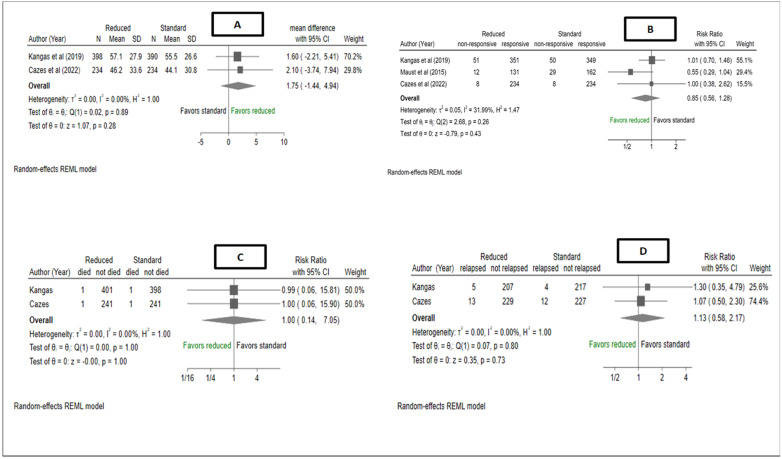
Forest plot of (**A**) time to recovery, (**B**) non-response rates, (**C**) mortality rates and (**D**) relapse rates from a meta-analysis of studies comparing reduced RUTF dose with standard RUTF dose for the treatment of severe wasting and/oedema. Effect estimates for Cazes et al. [14] (means and standard deviations) were obtained directly from the authors. Effect estimates for Kangas et al. [12] were computed from their open-source data. Kangas et al. [12], used length of stay, which was defined as number of days spent from admission to either recovery, referral, non-response, false discharge or last visit before defaulting. Non-response was assessed at 16 weeks in Kangas et al. [12] and 6 months in Cazes et al. [14]. Cazes et al. [14] used the time to nutritional improvement. Only ITT results were meta-analysed, for all studies.

Figure 5 shows a meta-analysis of time-to-recovery outcomes from the studies, with no observed difference in the mean time-to-recovery (days) between reduced RUTF dose and standard RUTF dose MD: 1.75 days, 95% CI (−1.44, 4.94), I^2^ = 0%, *p* = 0.28. A pooled estimate of non-response showed no differences in risk ratio between reduced dose RUTF and standard-dose RUTF, RR: 0.85, 95% CI (0.56, 1.28), I^2^ = 31.99%, *p* = 0.43. A pooled estimate for mortality found no difference in mortality risk ratio, 1.00, 95% CI (0.14, 7.05), I^2^ = 0%, *p* = 1, between reduced RUTF dose and standard RUTF dose groups. In the Maust et al. study, 21/326 (6%) children in the reduced dose arm were referred to inpatient treatment and/or died, whereas 29/537 (5%) of children in the standard arm were referred to inpatient treatment and/or died. These children were not followed up, and it could thus not be determined if they survived or not. There were no differences in relapses in the reduced RUTF dose group compared to the standard RUTF dose group (Figure 5), with a RR of 1.13, 95% CI (0.58, 2.17), I^2^ = 0%, *p* = 0.80.

In the findings by Kangas et al. [12], there were no differences in the number of children transferred to inpatient care between the reduced RUTF dose arm, 77/402 (19.2%), and the standard RUTF dose arm, 79/399 (20.1%). The mean difference was −0.7 (−6.2 to 4.8), *p* = 0.80. Similarly, the findings by Cazes et al. 2020 found no difference in children requiring inpatient care. In the reduced RUTF dose arm, 48/242 (20%) were referred for inpatient care, while 45/240 (19%) in the standard RUTF dose arm were referred, *p* = 0.85.

The overall risk of bias assessment for the studies showed a high risk of bias for Kangas et al. [12], and Maust et al. [13], and some concerns for the risk of bias for Cazes et al. [14] as shown in Supplementary Materials Figure S1. The high risk of bias in Kangas et al. was due to bias in the selection of reported results because of deviations from the pre-specified Statistical Analysis Plan (SAP).

## 4. Discussion

The systematic review included findings from three RCTs that were identified as eligible for inclusion to address the PICO question. The meta-analysis and narrative synthesis did not find differences between reduced RUTF doses and standard RUTF doses in treating severe wasting and/or oedema among 6–59-month-old children for most of the outcomes of interest. Only weight gain velocity between two weeks after initiation of treatment to discharge and rate of height gain was higher in the standard-dose RUTF group compared to reduced RUTF dose in the narrative synthesis of findings from Kangas et al. [12]. 

Although the findings of the meta-analysis show that reduced-dose RUTF and standard-dose RUTF have similar child health outcomes in treating severe wasting and/or oedema; it is important to recognise that anthropometric outcomes with the current WHO-recommended RUTF protocol remain suboptimal. For example, the rate of weight gain for either reduced RUTF dose or standard RUTF dose used in most national protocols does not meet the weight gain velocity of 5–10 g/kg/day recommended in the 1999 WHO guidelines [4] or the ≥8 g/kg/day as per the sphere standards [15] It remains unclear how realistic 1999 WHO guidelines are in most contexts and the Sphere standards are defined for humanitarian contexts which differ from the context of most programs. Therefore, although the lower doses may have similar outcomes to the WHO-recommended dose and may improve RUTF scalability, they may not improve the health outcomes of severely wasted children receiving treatment. 

The finding of a lower height gain velocity from admission to recovery with reduced RUTF dose compared to standard RUTF has limited inference as this outcome was only assessed in one study. There is a need for more studies to assess the robustness of this finding and the clinical significance of the reported difference. Further investigation of this finding is critical as children recovering from severe wasting and/or oedema have substantially poor linear growth than their healthy counterparts [16,17] and changes in RUTF dosing schemes should not worsen linear growth outcomes.

The effect sizes favouring the standard dose for the mean rate of weight gain, mean height, the mean rate of height gain, and WHZ at recovery observed only in the Maust et al. study could potentially be attributed to measurement errors in the standard arm. The Maust et al. study’s standard arm utilized non-research staff for both the delivery of therapeutic foods and the primary measurement of anthropometry, while the reduced dose arm for the same study and both arms in the Cazes et al. [14] and Kangas et al. [12] studies used research staff. Recognizing that anthropometric measurements are prone to measurement errors, quality control measures such as training, equipment calibration, and measurement standardization are typically employed in study conditions to promote accuracy [18,19,20]. In non-study conditions, similar levels of quality control are usually not employed and could lead to variation in results, as is also observed in the weekly rate of MUAC gain favoring the reduced dose arm in only the Maust et al. study.

The systematic review did not identify any studies that investigated higher RUTF doses; hence it remains unknown if higher RUTF doses are superior to the WHO recommended dose or lower doses. However, it is important to note that observed treatment with RUTF may also be attributable to a perceived suboptimal nutritional composition of RUTF. For example, there is evidence that the RUTF used in the included studies has a suboptimal composition for health outcomes in terms of micronutrients, essential fatty acids, iron and possibly protein quality [21,22,23]. Metabolic studies on energy requirements amongst children with severe wasting and/or oedema may provide better insights into the optimal doses of specific RUTF formulations required to achieve optimal recovery.

The main limitation of the systematic review is the low quality of evidence based on the high risk of bias for some of the studies and the low certainty of evidence. The systematic review was also not able to compare the impact of higher doses to the current WHO-recommended dose, as this was not reported in any of the screened studies. Despite the aforementioned limitations, the findings from this study are informative as this is the first quantitative evidence synthesis on the optimal dose and duration of RUTF in treating severe wasting and/or oedema. 

## 5. Conclusions

Based on the limited studies synthesized, there are no differences between reduced-dose RUTF and standard-dose RUTF in health outcomes when treating severe wasting and/or oedema among 6–59-month-old children. However, the evidence is insufficient and of limited certainty to make firm conclusions. More studies of better quality are required to resolve the question of the optimal dose and duration of RUTF for treating severe wasting and/or oedema amongst 6–59 months old children.

## Data Availability

All data used in this review were sourced from the published articles or directly from the authors of the individual studies.

## Supplementary Materials

The following supporting information can be downloaded at: https://www.mdpi.com/article/10.3390/nu15071750/s1, Figure S1: Risk of Bias; Table S1: Synthesis without Meta-analysis (SWiM) checklist for reporting items for a systematic review of varying doses and dose duration of Ready-to-use therapeutic food (RUTF) in the treatment of severe acute malnutrition (SAM); Table S2: Description of study outcomes; Table S3: Relevant ongoing studies for a systematic review of varying doses and dose duration of Ready-to-use therapeutic food (RUTF) in the treatment of severe acute malnutrition (SAM); Table S4: List of studies excluded full-text screening.
