# Supplementary material for: A Systematic Review on the Optimal Dose and Duration of Ready-to-Use Therapeutic Food (RUTF) for 6–59-Month-Old Children with Severe Wasting or Oedema
Source: Nutrients. 2023 Apr 3;15(7):1750. doi: 10.3390/nu15071750 (PMC10096907; doi:10.3390/nu15071750)

SUPPLEMENTARY MATERIALS

Supplementary Table S1

Synthesis Without Meta-analysis (SWiM) checklist for reporting items for a systematic review of varying doses and dose duration of Ready-to-use therapeutic food (RUTF) in the treatment of severe acute malnutrition (SAM)

**SWiM is intended to complement and be used as an extension to PRISMA**		
**SWiM reporting item**	**Item description**	**Page in manuscript where item is reported**
Methods		
**1** Grouping studies for synthesis	1a) Provide a description of, and rationale for, the groups used in the synthesis (e.g., groupings of populations, interventions, outcomes, study design)	Page 7
	1b) Detail and provide rationale for any changes made subsequent to the protocol in the groups used in the synthesis	Page 7
**2**.Describe the standardised metric and transformation methods used	Describe the standardised metric for each outcome. Explain why the metric(s) was chosen, and describe any methods used to transform the intervention effects, as reported in the study, to the standardised metric, citing any methodological guidance consulted	Page 7
**3**.Describe the synthesis methods	Describe and justify the methods used to synthesise the effects for each outcome when it was not possible to undertake a meta-analysis of effect estimates	Page 7
**4.** Criteria used to prioritise results for	Where applicable, provide the criteria used, with supporting justification, to select the studies, or a particular study, for the main synthesis or to draw conclusions from the synthesis (e.g.,	Page 7

summary and synthesis	based on study design, risk of bias assessments, directness in relation to the review question)	
**5**. Investigation of heterogeneity in reported effects	State the method(s) used to examine heterogeneity in reported effects when it was not possible to undertake a meta-analysis of effect estimates and its extensions to investigate heterogeneity	Page 7
**6** Certainty of evidence	Describe the methods used to assess certainty of the synthesis finding	Page 7
**7** Data presentation methods	Describe the graphical and tabular methods used to present the effects (e.g., tables, forest plots, harvest plots).<br>Specify key study characteristics (e.g., study design, risk of bias) used to order the studies, in the text and any tables or graphs, clearly referencing the studies included	Page 7
**8** Reporting results	For each comparison and outcome, provide a description of the synthesised findings, and the certainty of the findings. Describe the result in language that is consistent with the question the synthesis addresses, and indicate which studies contribute to the synthesis	Page 8 – 29
Discussion		
**9** Limitations of the synthesis	Report the limitations of the synthesis methods used and/or the groupings used in the synthesis, and how these affect the conclusions that can be drawn in relation to the original review question	Page 29

Overall search strategy and results of a systematic review on the optimal dose and duration of Ready-to-Use Therapeutic Food (RUTF) for 6–59-month-old children with severe wasting or oedema

**Database**	**Results**	**Date retrieved**
Ovid MEDLINE: Epub Ahead of Print, In-Process & Other Non-Indexed Citations, Ovid MEDLINE® Daily and Ovid MEDLINE® <1946-Preszent	824	09/09/2021
Web of science	1641	09/09/2021
CINAHL	2629	09/09/2021
Embase Classic+Embase <1947 to 2021 September 02>	1112	09/09/2021
COCHRANE	602	09/09/2021
Global Index Medicus	2430	09/09/2021
Total	9238	
Epistemonikos	342	28/09/2021
Clinicaltrials.gov	15	28/09/2021
WHO trials register	43	28/09/2021

Search results from Ovid MEDLINE: Epub Ahead of Print, In-Process & Other Non-Indexed Citations, Ovid MEDLINE® Daily and Ovid MEDLINE® <1946-Present>, for a systematic review on the optimal dose and duration of Ready-to-Use Therapeutic Food (RUTF) for 6–59-month-old children with severe wasting or oedema

	**Search terms**	**Results**
1	(RUTF or RTUF or plumpynut or yumplum or chiponde or purnavita or plumpyfield or diva-rutf or eezeepaste or edesia or proactiva or nourimanmba or dutasi or nutriset).tw, kw,ti,ab.	185
2	(("ready-to-use" or "ready to use") adj3 (food* or diet* or feed* or nutrient* or nutrition)).tw,kw,ti,ab.	339
3	(therapeutic adj3 (food* or diet* or feed* or nutrient* or nutrition)).tw,kw,ti,ab.	3015
4	1 or 2 or 3	3179
5	exp Infant/	1184969
6	exp Child, Preschool/	953799
7	(kindergarten* or kid* or toddler* or preschool* or pre-school* or infant* or child* or "Under-5s" or "Under 5s" or "Under 5" or baby or babies or boy* or girl* or pediatric* or paediatric*).tw,kw,ti,ab.	2633569
8	5 or 6 or 7	3283544
9	4 and 8	824

Search results from Ovid Embase (Embase Classic+Embase), for a systematic review on the optimal dose and duration of Ready-to-Use Therapeutic Food (RUTF) for 6–59-month-old children with severe wasting or oedema

	**Search term**	**Results**
1	exp "ready to use therapeutic food"/	93
2	(RUTF or RTUF or plumpynut or yumplum or chiponde or purnavita or plumpyfield or diva-rutf or eezeepaste or edesia or proactiva or nourimanmba or dutasi or nutriset).tw,kw,ti,ab.	210
3	(("ready-to-use" or "ready to use") adj3 (food* or diet* or feed* or nutrient* or nutrition)).tw,kw,ti,ab.	421
4	(therapeutic adj3 (food* or diet* or feed* or nutrient* or nutrition)).tw,kw,ti,ab.	4058
5	1 or 2 or 3 or 4	4308
6	exp infant/	1217414
7	exp preschool child/	652147
8	exp toddler/	5682
9	(kindergarten* or kid* or toddler* or preschool* or pre-school* or infant* or child* or "Under-5s" or "Under 5s" or "Under 5" or baby or babies or boy* or girl* or pediatric* or paediatric*).tw,kw,ti,ab.	3646820
10	6 or 7 or 8 or 9	4286895
11	5 and 10	1112

Search results from Web of science Core Collection, for a systematic review on the optimal dose and duration of Ready-to-Use Therapeutic Food (RUTF) for 6–59-month-old children with severe wasting or oedema

7	(#5) AND #6	1641
6	TS=((kindergarten* or kid* or toddler* or preschool* or pre-school* or infant* or child*))	3,396,756
5	(((#1) OR #2) OR #3) OR #4	8890
4	TS=(therapeutic NEAR/3 (food* or diet* or feed* or nutrient* or nutrition))	4185
3	TS=("ready to use" NEAR/3 (food* or diet* or feed* or nutrient* or nutrition))	4968
2	TS=("ready-to-use" NEAR/3 (food* or diet* or feed* or nutrient* or nutrition))	443
1	**TS=(RUTF or RTUF or plumpynut or yumplum or chiponde or purnavita or plumpyfield or diva-rutf or eezeepaste or edesia or proactiva or nourimanmba or dutasi or nutriset)**	192

Search results from Ebsco CINAHL Plus, for a systematic review on the optimal dose and duration of Ready-to-Use Therapeutic Food (RUTF) for 6–59-month-old children with severe wasting or oedema

#	Query	Limiters/Expanders	Last Run Via	Results
S25	S20 AND S24	Expanders - Apply equivalent subjects<br>Search modes - Boolean/Phrase	Interface - EBSCOhost Research Databases<br>Search Screen - Advanced Search<br>Database - CINAHL Plus with Full Text	2,629
S24	S21 OR S22 OR S23	Expanders - Apply equivalent subjects<br>Search modes - Boolean/Phrase	Interface - EBSCOhost Research Databases<br>Search Screen - Advanced Search<br>Database - CINAHL Plus with Full Text	1,961,054
S23	TX ((kindergarten* OR kid* OR toddler* OR preschool* OR pre-school* OR infant* OR child* OR "Under-5s" OR "Under 5s" OR "Under 5" OR baby OR babies OR boy* OR girl* OR pediatric* OR paediatric*)) OR TI ((kindergarten* OR kid* OR toddler* OR preschool* OR pre-school* OR infant* OR child* OR "Under-5s" OR "Under 5s" OR "Under 5" OR baby OR babies OR boy* OR girl* OR pediatric* OR paediatric*)) OR AB ((kindergarten* OR kid* OR toddler* OR preschool* OR pre-school* OR infant* OR child* OR "Under-5s" OR "Under 5s" OR "Under 5" OR baby OR babies OR boy* OR girl* OR pdiatrice* OR paediatric*))	Expanders - Apply equivalent subjects<br>Search modes - Boolean/Phrase	Interface - EBSCOhost Research Databases<br>Search Screen - Advanced Search<br>Database - CINAHL Plus with Full Text	1,961,054
S22	(MH "Child, Preschool")	Expanders - Apply equivalent subjects<br>Search modes - Boolean/Phrase	Interface - EBSCOhost Research Databases<br>Search Screen - Advanced Search	222,317

			Database - CINAHL Plus with Full Text	
S21	(MH "Infant+")	Expanders - Apply equivalent subjects<br>Search modes - Boolean/Phrase	Interface - EBSCOhost Research Databases<br>Search Screen - Advanced Search<br>Database - CINAHL Plus with Full Text	277,835
S20	S16 OR S17 OR S18 OR S19	Expanders - Apply equivalent subjects<br>Search modes - Boolean/Phrase	Interface - EBSCOhost Research Databases<br>Search Screen - Advanced Search<br>Database - CINAHL Plus with Full Text	7,402
S19	S1 OR S2 OR S3 OR S4 OR S5 OR S6 OR S7 OR S8 OR S9 OR S10 OR S11 OR S12 OR S13 OR S14 OR S15	Expanders - Apply equivalent subjects<br>Search modes - Boolean/Phrase	Interface - EBSCOhost Research Databases<br>Search Screen - Advanced Search<br>Database - CINAHL Plus with Full Text	201
S18	AB (("therapeutic" N3 (food* OR feed* OR diet* OR nutrient* OR nutrition))) OR TI (("therapeutic" N3 (food* OR feed* OR diet* OR nutrient* OR nutrition))) OR MW (("therapeutic" N3 (food* OR feed* OR diet* OR nutrient* OR nutrition))) OR TX (("therapeutic" N3 (food* OR feed* OR diet* OR nutrient* OR nutrition)))	Expanders - Apply equivalent subjects<br>Search modes - Boolean/Phrase	Interface - EBSCOhost Research Databases<br>Search Screen - Advanced Search<br>Database - CINAHL Plus with Full Text	7,137

S17	AB (("ready-to-use" N3 (food* OR feed* OR diet* OR nutrient* OR nutrition))) OR TI (("ready-to-use" N3 (food* OR feed* OR diet* OR nutrient* OR nutrition))) OR MW (("ready-to-use" N3 (food* OR feed* OR diet* OR nutrient* OR nutrition))) OR TX (("ready-to-use" N3 (food* OR feed* OR diet* OR nutrient* OR nutrition)))	Expanders - Apply equivalent subjects<br>Search modes - Boolean/Phrase	Interface - EBSCOhost Research Databases<br>Search Screen - Advanced Search<br>Database - CINAHL Plus with Full Text	299
S16	AB (("ready to use" N3 (food* OR feed* OR diet* OR nutrient* OR nutrition))) OR TI (("ready to use" N3 (food* OR feed* OR diet* OR nutrient* OR nutrition))) OR MW (("ready to use" N3 (food* OR feed* OR diet* OR nutrient* OR nutrition))) OR TX (("ready to use" N3 (food* OR feed* OR diet* OR nutrient* OR nutrition)))	Expanders - Apply equivalent subjects<br>Search modes - Boolean/Phrase	Interface - EBSCOhost Research Databases<br>Search Screen - Advanced Search<br>Database - CINAHL Plus with Full Text	340
S15	AB nutriset OR TI nutriset OR MW nutriset OR TX nutriset	Expanders - Apply equivalent subjects<br>Search modes - Boolean/Phrase	Interface - EBSCOhost Research Databases<br>Search Screen - Advanced Search<br>Database - CINAHL Plus with Full Text	34
S14	AB dutasi OR TI dutasi OR MW dutasi OR TX dutasi	Expanders - Apply equivalent subjects<br>Search modes - Boolean/Phrase	Interface - EBSCOhost Research Databases<br>Search Screen - Advanced Search<br>Database - CINAHL Plus with Full Text	0
S13	AB nourimanmba OR TI nourimanmba OR MW nourimanmba OR TX nourimanmba	Expanders - Apply equivalent subjects<br>Search modes - Boolean/Phrase	Interface - EBSCOhost Research Databases<br>Search Screen - Advanced Search<br>Database - CINAHL Plus with Full Text	0
S12	AB proactiva OR TI proactiva OR MW proactiva OR TX proactiva	Expanders - Apply equivalent subjects<br>Search modes - Boolean/Phrase	Interface - EBSCOhost Research Databases<br>Search Screen - Advanced Search	55

			Database - CINAHL Plus with Full Text	
S11	AB edesia OR TI edesia OR MW edesia OR TX edesia	Expanders - Apply equivalent subjects<br>Search modes - Boolean/Phrase	Interface - EBSCOhost Research Databases<br>Search Screen - Advanced Search<br>Database - CINAHL Plus with Full Text	5
S10	AB edesia OR TI edesia OR MW edesia OR TX edesia	Expanders - Apply equivalent subjects<br>Search modes - Boolean/Phrase	Interface - EBSCOhost Research Databases<br>Search Screen - Advanced Search<br>Database - CINAHL Plus with Full Text	5
S9	AB eezeepaste OR TI eezeepaste OR MW eezeepaste OR TX eezeepaste	Expanders - Apply equivalent subjects<br>Search modes - Boolean/Phrase	Interface - EBSCOhost Research Databases<br>Search Screen - Advanced Search<br>Database - CINAHL Plus with Full Text	0
S8	AB diva-rutf OR TI diva-rutf OR MW diva-rutf OR TX diva-rutf	Expanders - Apply equivalent subjects<br>Search modes - Boolean/Phrase	Interface - EBSCOhost Research Databases<br>Search Screen - Advanced Search<br>Database - CINAHL Plus with Full Text	0
S7	AB plumpyfield OR TI plumpyfield OR MW plumpyfield OR TX plumpyfield	Expanders - Apply equivalent subjects<br>Search modes - Boolean/Phrase	Interface - EBSCOhost Research Databases<br>Search Screen - Advanced Search<br>Database - CINAHL Plus with Full Text	0
S6	AB purnavita OR TI purnavita OR MW purnavita OR TX purnavita	Expanders - Apply equivalent subjects<br>Search modes - Boolean/Phrase	Interface - EBSCOhost Research Databases<br>Search Screen - Advanced Search	0

			Database - CINAHL Plus with Full Text	
S5	AB chiponde OR TI chiponde OR MW chiponde OR TX chiponde	Expanders - Apply equivalent subjects<br>Search modes - Boolean/Phrase	Interface - EBSCOhost Research Databases<br>Search Screen - Advanced Search<br>Database - CINAHL Plus with Full Text	1
S4	AB chiponde OR TI chiponde OR MW chiponde OR TX chiponde	Expanders - Apply equivalent subjects<br>Search modes - Boolean/Phrase	Interface - EBSCOhost Research Databases<br>Search Screen - Advanced Search<br>Database - CINAHL Plus with Full Text	1
S3	AB yumplum OR TI yumplum OR MW yumplum OR TX yumplum	Expanders - Apply equivalent subjects<br>Search modes - Boolean/Phrase	Interface - EBSCOhost Research Databases<br>Search Screen - Advanced Search<br>Database - CINAHL Plus with Full Text	0
S2	AB plumpynut OR TI plumpynut OR MW plumpynut OR TX plumpynut	Expanders - Apply equivalent subjects<br>Search modes - Boolean/Phrase	Interface - EBSCOhost Research Databases<br>Search Screen - Advanced Search<br>Database - CINAHL Plus with Full Text	1
S1	AB RUTF OR TI RUTF OR MW RUTF OR TX RUTF	Expanders - Apply equivalent subjects<br>Search modes - Boolean/Phrase	Interface - EBSCOhost Research Databases<br>Search Screen - Advanced Search<br>Database - CINAHL Plus with Full Text	109

Search results from Cochrane library for a systematic review on the optimal dose and duration of Ready-to-Use Therapeutic Food (RUTF) for 6–59-month-old children with severe wasting or oedema

#	Search	results
#1	(RUTF or RTUF or plumpynut or yumplum or chiponde or purnavita or plumpyfield or diva-rutf or eezeepaste or edesia or proactiva or nourimanmba or dutasi or nutriset):ti,ab,kw	162
#2	("ready to use" NEAR/3 (food* OR feed* OR diet* OR nutrient* OR nutrition)):ti,ab,kw	268
#3	(((ready-to-use) NEAR/3 (food* OR feed* OR diet* OR nutrient* OR nutrition))):ti,ab,kw	247
#4	(therapeutic NEAR/3 (food* OR feed* OR diet* OR nutrient* OR nutrition)):ti,ab,kw	1645
#5	#1 OR #2 OR #3 OR #4	1815
#6	MeSH descriptor: [Child] explode all trees	58448
#7	MeSH descriptor: [Infant] explode all trees	33346
#8	(kindergarten* or kid* or toddler* or preschool* or pre-school* or infant* or child* or "Under-5s" or "Under 5s" or "Under 5" or baby or babies or boy* or girl* or pediatric* or paediatric*):ti,ab,kw	261882
#9	#6 OR #7 OR #8	261882
#10	#5 AND #9	602

Search results from Global Index Medicus for a systematic review on the optimal dose and duration of Ready-to-Use Therapeutic Food (RUTF) for 6–59-month-old children with severe wasting or oedema

**Search #**	**Search**	**results**
1	(tw:(rutf OR rtuf OR plumpynut OR yumplum OR chiponde OR purnavita OR plumpyfield OR diva-rutf OR eezeepaste OR edesia OR proactiva OR nourimanmba OR dutasi OR nutriset))	**97**
2	(tw:("ready to use" AND (food* OR diet* OR feed* OR nutrient* OR nutrition)))	165
3	(tw:(ready-to-use AND (food* OR diet* OR feed* OR nutrient* OR nutrition)))	166
4	(tw:(therapeutic AND (food* OR diet* OR feed* OR nutrient* OR nutrition)))	8138
5	(tw:(kindergarten* or kid* or toddler* or preschool* or pre-school* or infant* or child* or "Under-5s" or "Under 5s" or "Under 5" or baby or babies or boy* or girl* or pediatric* or paediatric*))	**513077**
6	(tw:((tw:((kindergarten* or kid* or toddler* or preschool* or pre-school* or infant* or child* or "Under-5s" or "Under 5s" or "Under 5" or baby or babies or boy* or girl* or pediatric* or paediatric*))))) AND (tw:((((tw:(rutf OR rtuf OR plumpynut OR yumplum OR chiponde OR purnavita OR plumpyfield OR diva-rutf OR eezeepaste OR edesia OR proactiva OR nourimanmba OR dutasi OR nutriset)) OR (tw:("ready TO use" AND (food* OR diet* OR feed* OR nutrient* OR nutrition))) OR (tw:("ready-to-use" AND (food* OR diet* OR feed* OR nutrient* OR nutrition))) OR (tw:(therapeutic AND (food* OR diet* OR feed* OR nutrient* OR nutrition))))))	**2629**

Supplementary Table S2

Main outcome	Description of the outcome
Anthropometric recovery	Participants who attain a MUAC of at least 12.5 cm or WHZ of at least -2 SD, and no oedema for at least two weeks or based on the author's definition.
Anthropometric outcomes	WLZ/WHZ, weight-for-age Z scores (WAZ), MUAC, change in anthropometry, weight gain
Sustained recovery	Participants who achieve sustained recovery in a prespecified period e.g., Recovery that lasts more than 6 months (author-based definition)
Time to recovery	Time taken to reach a MUAC of at least 12.5 cm or WHZ of at least -2 SD, and no oedema for at least two weeks or based on the author-definition
Non-response	Participants who fail to achieve recovery within a specified time (author-based definition).
Mortality	Percentage of infants and children who died
Readmission	Requiring treatment for severe wasting in an inpatient or outpatient/community setting within a specific period e.g., six months after discharge (author-based definition)

Supplementary Table S3

Relevant ongoing studies for a systematic review of varying doses and dose duration of Ready-to-use therapeutic food (RUTF) in the treatment of severe acute malnutrition (SAM)

**Study name**	**Optimizing Acute Malnutrition Management in Children Aged 6 to 59 Months in Niger (OptiMA Niger)**	**Severe acute malnutrition treatment delivered by community health workers in emergency settings of Mali (iCCM+ Project)**	**Effectiveness of a reduced dose of ready-to-use therapeutic food (RUTF) in the management of uncomplicated severe acute malnutrition (SAM)**
**Study design**	Randomised clinical intervention trial	Cluster randomized controlled non-inferiority trial (Treatment)	Retrospective randomised, controlled, non-inferiority trial in real-life conditions
**Participants**	Children aged 6 -59 months	Children aged 6 -59 months diagnosed with uncomplicated severe acute malnutrition	Children aged 6-59 months with medically uncomplicated severe acute malnutrition
**Intervention**	**Arm 1 OptiMA**<br>RUTF 170 kcal/kg/d for children with nutritional edema or MUAC < 115 mm; 125 kcal/kg/d for MUAC 115-119 mm and 75 kcal/kg/d for MUAC 120-124 mm.<br>**Arm 2 ComPAS**<br>RUTF 1000 kcal/d for children with nutritional oedema or MUAC < 115 mm and 500 kcal/day for MUAC 115-124 mm.	**Arm 1** treatment is provided in health centres and outside by community health workers following national protocol.<br><u>Admission criteria</u>: Oedema +/++ and/or WHZ <-3 score and/or MUAC <115mm.<br>Treatment: RUTF according to weight (170 Kcal/kg/day). <u>Discharge criteria</u>: WHZ > -1.5 score or MUAC >=125 mm.<br><br>**Arm 2** treatment is provided in health centres and outside them by community health workers following a modified protocol.<br>Admission criteria: Oedema +/++ and/or MUAC <115mm.<br>Treatment: a fixed amount of 2 sachets of RUTF /day (1000 Kcal/day) except children under 5Kg who will receive 1 sachet/day (500Kcal/day).<br>Discharge criteria: WHZ >-1.5 z-score or MUAC >=125mm.	Children will receive RUTF in the standard dose for the first 2 weeks. From the third week onwards, it will be reduced according to their weight.

**Study name**	**Optimizing Acute Malnutrition Management in Children Aged 6 to 59 Months in Niger (OptiMA Niger)**	**Severe acute malnutrition treatment delivered by community health workers in emergency settings of Mali (iCCM+ Project)**	**Effectiveness of a reduced dose of ready-to-use therapeutic food (RUTF) in the management of uncomplicated severe acute malnutrition (SAM)**
**Comparator**	**Standard**<br>RUTF 130-200 kcal/kg/day for children with nutritional oedema or MUAC < 115 mm or WHZ <-3 and RUSF 500 kcal/d for children 6-23 months with WHZ between -2 and -3 Z and MUAC 115-124 mm.	Treatment is provided only in health centres following the national protocol.<br>Admission criteria: Oedema +/++ and/or WHZ <-3 z-score and/or MUAC <115mm.<br>Treatment: RUTF according to weight (170 Kcal/kg/day).<br>Discharge criteria: WHZ >-1.5 z-score or MUAC >=125 mm.	Children will receive the standard RUTF dose from the first week onwards
**Outcomes**	Primary:<br>i. success rate<br>Secondary<br>i. Recovery rate in children with SAM WHO definition<br>ii. Recovery rate in children with MUAC < 115 mm<br>Other outcomes<br>i. Cost-effectiveness of OptiMA and ComPAS strategies<br>ii. Outpatient recovery rate<br>iii. Consumption of RUTF<br>iv. Recovered children by RUTF<br>v. Relapse rate to a new episode<br>vi. Non-response rate<br>vii. Recovery rate of children without supplementation<br>viii. Recovery rate of WaST children<br>ix. · Hospitalisation rate	Primary<br>i. Recovery rate<br>ii. Default rate<br>iii. Deceased rate<br>iv. Referral rate<br>Secondary<br>i. Coverage compared at baseline and end-line<br>ii. Cost-effectiveness<br>iii. Severity at admission<br>iv. Recovery time<br>v. Number of follow up<br>vi. Number of RUTF<br>vii. Average weight and MUAC gain of those recovered<br>viii. Number of cases treated for other non-severe common diseases	Primary<br>i. Weight gain velocity<br>Secondary<br>i. Acceptance of a reduced dose during SAM treatment by health care personnel and the community<br>ii. Relapse rate<br>iii. Psychomotor development<br>iv. Duration of oedema<br>v. Length of stay<br>vi. Cure rate<br>vii. Dropout rate<br>viii. Death rate<br>ix. Cost savings<br>x. Prevalence of stunting
**Start date**	22 March 2021	1st July 2020	1st February 2021
**End date**	June 2022	30 October 2021	30th July 2022

**Study name**	**Optimizing Acute Malnutrition Management in Children Aged 6 to 59 Months in Niger (OptiMA Niger)**	**Severe acute malnutrition treatment delivered by community health workers in emergency settings of Mali (iCCM+ Project)**	**Effectiveness of a reduced dose of ready-to-use therapeutic food (RUTF) in the management of uncomplicated severe acute malnutrition (SAM)**
**Contact information**	Contact: Maguy Daures, MSc +33557571539 maguy.daures@coral.alima.ngo Contact: Hien Jérémie, MD, MPH +33557571539 zinder.cpoptima@niger.alima.ngo	Name: Noemi Lopez-Ejeda Address: Action Against Hunger Spain C/ Duque de Sevilla 3. C.P. 28002 Madrid Spain Telephone: +34 91 391 53 00 Email: nlopez@accioncontraelhambre.org	Julien Ntaongo Alendi Address: Campus Université de Kinshasa Kinshasa, BP 127 Kinshasa XI, Congo Democratic Republic Telephone: +243 826 083 359 Email: jntaongo@gmail.com

<u>Supplementary Table S4</u>

List of studies excluded full-text screening

	Author	Reasons for exclusion
1	(Shepherd and Becquet 2018)	This was a study protocol registration on the Optimising acute MAlnutrition (OptiMA) trial, thus, did not include any results. However, the preprint is included in the review (Cazes preprint).
2	(Chase et al. 2020)	The study design was ineligible because it was an observational study that lacked a comparison group.
3	(Aguayo et al. 2013)	The study design was ineligible because it was an observational study that lacked a comparison group.
4	(Lelijveld et al. 2018)	This was a study protocol of the cost-effectiveness component in the ComPAS trial, thus, did not include any results on the relevant outcomes.
5	(Isanaka et al. 2009)	The study was ineligible because it did not include the study population of interest i.e., children with SAM, based on wasting, and/or oedema.
6	(James et al. 2015)	The study design was ineligible because it was a retrospective analysis of patient records and lacked a comparison group.
7	(Daures et al. 2020)	The study design was ineligible because it did not have a comparison group; this was a single-arm OptiMA-proof-of-concept trial, which aimed to assess if the outcomes met the Sphere standards.
8	(Stephenson et al. 2021)	The study design was ineligible because it was not a randomised controlled trial; this was a dual-cohort study that was a secondary analysis of data from 2 different clinical trials (each trial contributed one arm).
9	(Kangas et al. 2020)	The study was ineligible because it was a subsequent paper to the study by Kangas 2019, that was already included in this review and did not report any new information on the outcomes of interest.
10	(Nikiema et al. 2021)	The study was ineligible because it was a subsequent paper to the study by Kangas 2019 that was already included in this review and did not report any new information on the outcomes of interest. This study focused on nutrient intakes of the same study participants as the included study
11	(Hossain et al. 2009)	The intervention and comparator were ineligible because they did not contain the treatment of interest (RUTF).

	Author	Reasons for exclusion
12	(Brewster, Manary, and Graham 1997)	The intervention was ineligible because it did not contain the treatment of interest (RUTF). This study was a comparison of routine tube feeding and micronutrient supplementation against no tube feeding in children in NRUs with oedematous malnutrition
13	(Khan et al. 2021)	The intervention was ineligible because it used 50% of RUTF and 50% of home-based food (intervention group 1) and 100% home-based food only (intervention group 2). The full study results could not be obtained from the authors.
14	(Khan 2020)	This was a study protocol registration, as such it did not include any results. An abstract which reported the trial results from this protocol was also excluded (See Khan et al 2021 study number 14)
15	(Bellows et al. 2009)	The intervention was ineligible because it did not contain the treatment of interest (RUTF) as an intervention.
16	(Bailey et al. 2020)	The comparator was ineligible because it used RUSF in children who progressed from SAM to MAM, which is contrary to the WHO-definition of standard treatment

Supplementary Figure S1

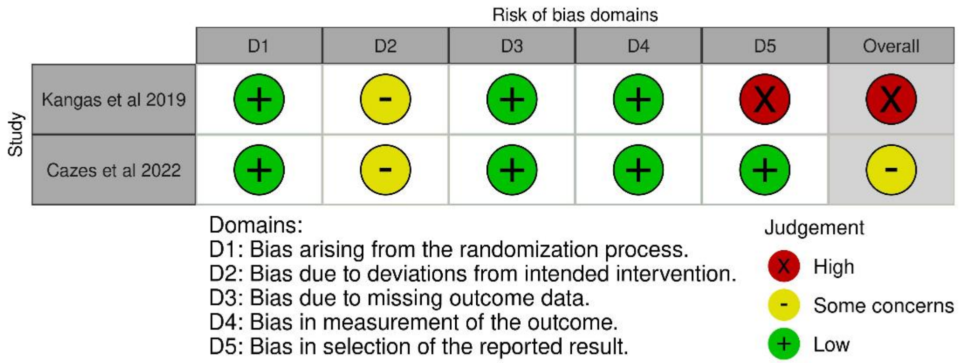

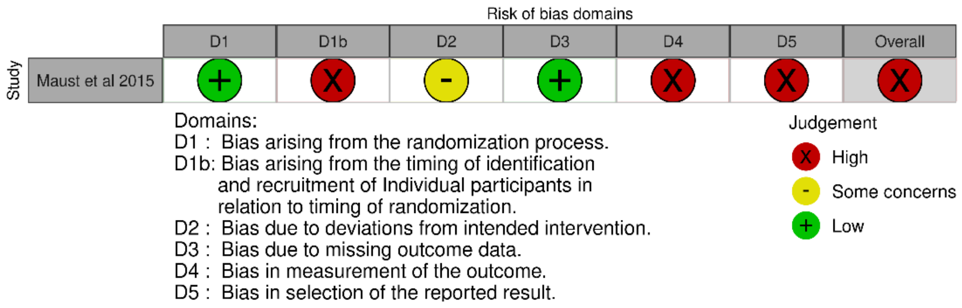

Supplement: Supplementary file 1 [file nutrients-15-01750-s001.zip › nutrients-2275565-supplementary.pdf]
